# Distinct and Overlapping Expression Patterns of the Homer Family of Scaffolding Proteins and Their Encoding Genes in Developing Murine Cephalic Tissues

**DOI:** 10.3390/ijms21041264

**Published:** 2020-02-13

**Authors:** Claes-Göran Reibring, Kristina Hallberg, Anders Linde, Amel Gritli-Linde

**Affiliations:** 1 Department of Oral Biochemistry, Institute of Odontology, Sahlgrenska Academy at the University of Gothenburg, SE-40530 Göteborg, Sweden; claes-goran.reibring@gu.se (C.-G.R.); kristina.hallberg@odontologi.gu.se (K.H.); linde@odontologi.gu.se (A.L.); 2 Public Dental Service, Region Västra Götaland, SE-45131 Uddevalla, Sweden

**Keywords:** ameloblasts, choroid plexus, cochlea, endothelium, hypothalamus, hippocampal formation, odontoblasts, olfactory epithelium, salivary glands, taste buds

## Abstract

In mammals Homer1, Homer2 and Homer3 constitute a family of scaffolding proteins with key roles in Ca^2+^ signaling and Ca^2+^ transport. In rodents, Homer proteins and mRNAs have been shown to be expressed in various postnatal tissues and to be enriched in brain. However, whether the Homers are expressed in developing tissues is hitherto largely unknown. In this work, we used immunohistochemistry and in situ hybridization to analyze the expression patterns of Homer1, Homer2 and Homer3 in developing cephalic structures. Our study revealed that the three Homer proteins and their encoding genes are expressed in a wide range of developing tissues and organs, including the brain, tooth, eye, cochlea, salivary glands, olfactory and respiratory mucosae, bone and taste buds. We show that although overall the three Homers exhibit overlapping distribution patterns, the proteins localize at distinct subcellular domains in several cell types, that in both undifferentiated and differentiated cells Homer proteins are concentrated in puncta and that the vascular endothelium is enriched with Homer3 mRNA and protein. Our findings suggest that Homer proteins may have differential and overlapping functions and are expected to be of value for future research aiming at deciphering the roles of Homer proteins during embryonic development.

## 1. Introduction

The Homer family of scaffolding proteins is highly conserved across species, and in mammals it consists of three members called Homer1, Homer2 and Homer3 encoded by three different genes *Homer1*, *Homer2*/*Cupidin* and *Homer3* [1,2,3,4,5]. In mammals alternative RNA splicing generates several Homer isoforms which are sub-classified into short and long forms [3,5,6,7,8]. Homer proteins exhibit a highly conserved N-terminal domain known as Enabled/vasodilator-stimulated phosphoprotein (Ena/VASP) homology 1 (EVH1) domain. This domain binds to PPXXF, øPPXF or LPSSP consensus motifs [9,10,11] found in target proteins, including proteins involved in Ca^2+^ signaling such as group I metabotropic glutamate receptors1/5 (mGluR1/5), inositol 1,4,5-triphosphate receptors (IP3Rs), transient receptor potential canonical (TRPC) channels, ryanodine receptors (RyRs) and phospholipase Cβ [1,7,12,13,14]. Unlike the short Homer isoforms, which display a truncated C-terminal domain, the long Homer isoforms have a long C-terminal domain comprising a coiled-coil domain with leucine zipper motifs. The coiled-coil domain enables the long Homer proteins to self-associate or to associate with other Homer family members, thereby promoting the formation of homo- and hetero-multimers [3,6,7,15,16,17]. The Mouse *Homer1a* [3] alias *vesl-1S* [6] transcript variant encodes a short Homer1 protein and is a neuronal immediate-early gene induced by neuronal activity [1,2,3]. It has been shown that in stimulated neurons, Homer1a acts in a dominant-negative manner, blocking the scaffolding ability of long Homer isoforms and altering their signaling [7,12].

The long Homer isoforms, including *Homer1b* and *Homer1c* [3], also known as *vesl-1L* [6] and *PSD-Zip45* [4,15], respectively, as well as *Homer2a*/*vesl-2*/*Cupidinα, Homer2b*/*vesl-2*/*Cupidin*β, *Homer3a* and *Homer3b* [3,5,6,7,18] are constitutively expressed in brain [3,4,7,18].

In the rodent brain, the long forms of Homer proteins perform various functions, including modulation of Ca^2+^ signaling by assembling macromolecular complexes through binding to other postsynaptic density proteins such as mGluR1/5, IP_3_Rs and several postsynaptic scaffolding proteins [7,17,19]. Other functions of Homer proteins in the central nervous system include regulation of axonal pathfinding [20], regulation of dendritic arborization and branching through IP3R signaling [21] and control of trafficking and anchoring of mGluR1/5 [7]. 

Loss-of-function or overexpression of Homer genes in rodents revealed roles for these proteins in neuropsychological disorders, ranging from impairment of memory and motivation-based learning tasks to compulsive behavior and addiction to drugs and alcohol [7,22,23,24] and clinical studies implicated Homer dysfunction in the pathogenesis of depression, schizophrenia and addiction [25,26,27]. 

Homer transcripts and/or proteins have also been shown to be expressed in adult non-neuronal tissues such as skeletal muscle [7,28,29,30,31], thymus, heart, kidney [7], pancreas [32] and salivary glands [33]. Homer1 loss-of-function in mice causes skeletal myopathy [34] and humans and mice with Homer2 loss-of-function suffer from impaired hearing and progressive hearing loss, respectively [35].

In addition to binding and modulating the activities of IP3Rs, RyRs and TRPC channels [7,8,14,36], Homer proteins also interact physically and/or functionally with other Ca^2+^-handling proteins, such as the plasma-membrane Ca^2+^-ATPase (PMCA) pumps [33,37,38] as well as with Orai1 and Stromal interaction molecule 1 (Stim1), key proteins of the store-operated Ca^2+^ entry (SOCE) system [39,40], a ubiquitous physiological process with a crucial role in the regulation of extracellular Ca^2+^ influx into cells and a major mechanism of Ca^2+^ influx in non-electrically excitable cells [8,41]. 

In developing rodent teeth, it has been shown that PMCA pumps and their encoding genes [42] as well as Orai and Stim proteins and their encoding genes [43,44,45,46,47] are expressed in ameloblasts, the enamel-forming cells, and genetic studies in humans and mice have revealed a critical role for Orai and Stim proteins for proper enamel formation [43,46,47,48,49]. 

These findings prompted us to determine whether Homer proteins and their encoding genes are expressed in murine developing teeth. In addition, since whether Homer1, Homer2 and Homer3 are expressed in developing tissues is largely unknown, we analyzed their expression at the mRNA and/or protein levels in several developing murine cephalic structures, including the brain, olfactory and respiratory mucosae, cochlea, taste buds, salivary glands and bone. We found that all three Homer family members are not only produced in cells and tissues performing highly specialized and metabolically-demanding functions, such as secretion, sensory perception, as well as formation or degradation of extracellular matrices but also in undifferentiated cells during growth and morphogenesis of organs, including the tooth and salivary glands. Furthermore, our study revealed that while the Homers generally exhibit overlapping expression patterns, in several cell types the proteins localize at distinct subcellular domains, that in a multitude of tissues Homer proteins are conspicuously enriched in intracellular puncta and that the endothelium of blood vessels in cephalic tissues and organs, including the brain, produces all three Homer family members and is enriched with Homer3. 

## 2. Results

### 2.1. Specificities of Homer Probes and Anti-Homer Antibodies 

To test the specificities of the probes targeting *Homer1*, *Homer2* and *Homer3* transcripts, we carried out in situ hybridization on sections across the postnatal mouse hippocampus. Consistent with previous findings [3,4] (see also https://mouse.brain-map.org), we found that *Homer1*, *Homer2* and *Homer3* transcripts were enriched in the hippocampal regions CA1, CA2 and CA3, respectively (Figure S1A–C). The specificities of the affinity-purified Homer1, Homer2 and Homer3 antibodies used in this study have been tested and validated by the manufacturers. As an additional antibody specificity test, we immunostained sections across the hippocampus and found that the distribution patterns of Homer1, Homer2 and Homer3 proteins (Figure S1D–F’) were consistent with those found previously using different antibodies targeting these proteins [15,18]. It is noteworthy that the distribution of *Homer* transcripts differs slightly from that of Homer proteins (Figure S1A–F’) and this is expected, as prior work showed that in various brain regions, including the hippocampus, *Homer* transcripts are confined to neuronal somata [3,4] (see also https://mouse.brain-map.org), whereas the proteins are in general detectable in both neuronal somata and axons/dendrites [3,5,7,15,18]. 

As described below, we found that the distribution patterns of Homer1, Homer2 and Homer3 proteins in various cephalic tissues and cells (except neuronal processes and nerves) faithfully recapitulated the expression patterns of their encoding transcripts. Furthermore, red blood cells were devoid of hybridization signals and immunostaining for the three Homer family members, constituting reliable internal negative controls. Finally, no immunostaining was detected upon omission of the primary antibodies (Figure S1G,H). Taken together, these findings are strong evidence of the specificities of the probes and antibodies used in this study.

### 2.2. Expression Patterns of Homer1, Homer2 and Homer3 in the Developing Tooth 

The distribution patterns of Homer1, Homer2 and Homer3 proteins during tooth development are summarized in Table S1.

Developing teeth undergo a series of well-defined, sequential stages during which the dental epithelium and the neural crest-derived dental mesenchyme interact to drive tooth formation (odontogenesis) from initiation until completion [50,51]. We found that all three Homer family members are expressed during odontogenesis. 

At embryonic day 12.5 (E12.5), development of a localized thickening of the oral epithelium, known as the dental placode, marks initiation of tooth formation. By E13.5 (bud stage of odontogenesis) the dental epithelium has grown and invaginated into the developing jaw to form a bud surrounded by the dental mesenchyme. At E14.5 (cap stage of odontogenesis), the epithelial bud forms a cap-shaped structure encompassing the dental mesenchyme (dental papilla).

**Figure 1:**
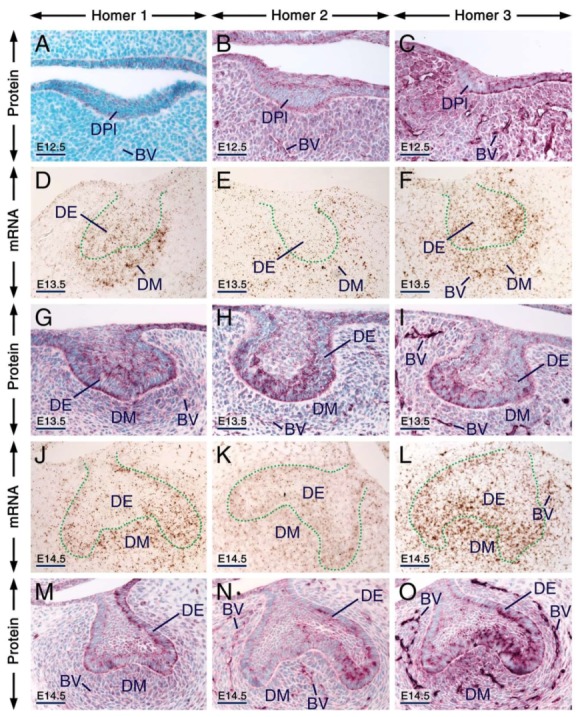
Expression patterns of Homer1, Homer2 and Homer3 during early stages of tooth formation. (**A**–**C**) Representative immunostaining of frontal sections across the first molars at embryonic day 12.5 (E12.5) showing the distribution of Homer1 (**A**), Homer2 (**B**) and Homer3 (**C**) proteins (purple). All three Homer proteins are expressed in the dental placode (epithelium) and Homer1 protein is concentrated in puncta. The jaw mesenchyme, including the mesenchyme adjacent to the dental placode, expresses Homer2 and Homer3 proteins and the vascular endothelium shows weak Homer1, moderate Homer2 and strong Homer3 immunostaining. (**D**–**O**) Representative in situ hybridization (**D**–**F**,**J**–**L**) and immunohistochemistry (**G**–**I**,**M**–**O**) data in frontal (**D**–**F**,**J**–**O**) and parasagittal (**G**–**I**) sections across the first molars at E13.5 (**D**–**I**; bud stage) and E14.5 (**J**–**O**; cap stage) showing expression of *Homer1*, *Homer2* and *Homer3* transcripts (brown) and Homer1, Homer2 and Homer3 proteins (purple) in the dental epithelium and dental mesenchyme. Cells of the dental epithelium exhibit diffuse Homer immunostaining of the cytoplasm and membranes as well as puncta enriched with Homer proteins. The vascular endothelium is enriched with Homer3 protein and mRNA and exhibits weak Homer1 and moderate Homer2 immunostaining. The dotted line in (**D**–**F**) and (**J**–**L**) highlights the junction between the dental epithelium and the dental mesenchyme. BV, blood vessels; DE, dental epithelium; DPl, dental placode; DM, dental mesenchyme. Scale bars: 50 µm (**A**–**O**).

At E12.5, all three Homer proteins were detectable in the dental placode and the mesenchyme underlying the placode showed immunostaining for Homer2 and Homer3 but not for Homer1 (Figure 1A–C). Remarkably, in cells of the dental placode Homer1 and to a lesser extent Homer2 and Homer3, immunostaining was concentrated in puncta (Figure 1A–C). At E13.5 and E14.5, Homer1, Homer2 and Homer3 mRNAs and proteins were readily detectable in the dental epithelium and dental mesenchyme (Figure 1D–O). At these stages, subsets of cells of the dental epithelium displayed puncta enriched with Homer1, Homer2 and Homer3 proteins (Figure 1G–I,M–O). At E12.5 (Figure 1A–C), E13.5 (Figure 1G–I) and E14.5 (Figure 1M–O), cells (likely endothelial cells) lining the internal surface of the wall of blood vessels in the dental mesenchyme and the mesenchyme of the developing jaws showed weak anti-Homer1, moderate anti-Homer2 and strong anti-Homer3 immunoreactivities.

At 1 day post-partum (1 dpp; bell stage of odontogenesis), cusp morphogenesis in molars is underway and the various cell types of the dental epithelium (also called enamel organ) and dental papilla are histologically distinguishable. The enamel organ comprises the outer dental epithelium, the stellate reticulum, the stratum intermedium and the inner dental epithelium. The latter comprises proliferating ameloblast precursor cells adjacent to the dental papilla [51,52]. In the dental papilla, preodontoblasts, cells that are adjacent to the inner dental epithelium, differentiate into odontoblasts which at this stage begin to produce predentin matrix, whereas dental papilla cells that are away from the inner dental epithelium form the dental pulp [53]. Cells of the inner dental epithelium adjacent to the predentin matrix cease to proliferate and initiate differentiation (differentiating ameloblasts) [52,53]. In molars, odontoblast and ameloblast cytodifferentiation begins first in the main cusp and the wave of cytodifferentiation, which in a given cusp occurs progressively from the tip of the cusp to the future crown-root junction, continues until tooth crown formation is completed [52,54]. 

**Figure 2:**
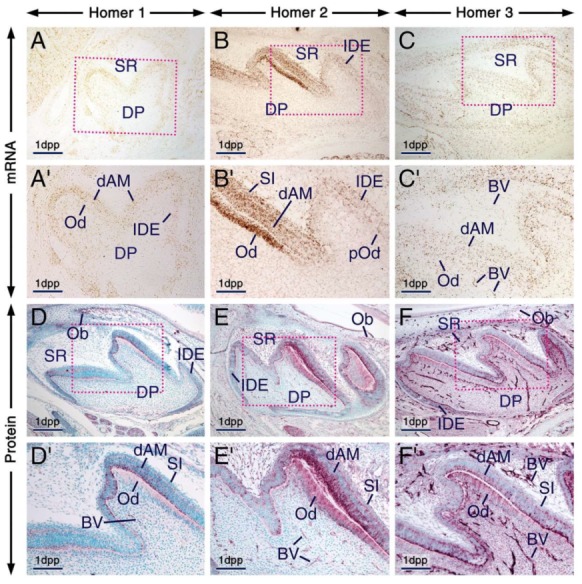
Expression patterns of Homer1, Homer2 and Homer3 during the bell stage of tooth development. (**A**–**F**) Representative frontal (**A**) and parasagittal (**B**–**F**) sections across developing first molars at 1 day postpartum (1 dpp) after in situ hybridization (**A**–**C**) and immunohistochemistry (**D**–**F**) for Homer1 (**A**,**D**), Homer2 (**B**,**E**) and Homer3 (**C**,**F**). (**A’**–**F’**) are magnified views of the boxed areas in (**A**–**F**). Odontoblasts and differentiating ameloblasts are enriched with Homer proteins (purple) and mRNAs (brown) as compared to the dental papilla, stellate reticulum and inner dental epithelium. Homer2 protein and mRNA are also expressed in the stratum intermedium. Homer proteins are concentrated in intracellular puncta in odontoblasts and differentiating ameloblasts as well as in cells of the stratum intermedium and inner dental epithelium. The endothelium of blood vessels in the stellate reticulum and dental mesenchyme is enriched with Homer3 mRNA and protein and exhibits weak and moderate immunostaining for Homer1 and Homer2, respectively. Homer proteins are also detectable in osteoblasts within the developing alveolar bone (**D**–**F**). BV, blood vessels; dAM, differentiating ameloblasts; DP, dental papilla; IDE, inner dental epithelium; Ob, osteoblasts; Od, odontoblasts; pOd, preodontoblasts; SI, stratum intermedium; SR, stellate reticulum. Scale bars: 200 µm (**A**–**F**) and 100 µm (**A’**–**F’**).

At 1 dpp, immunolabelling and hybridization signals for the three Homers in odontoblasts and differentiating ameloblasts were stronger than in other components of the enamel organ and dental papilla, one exception, however, being the stratum intermedium which was enriched with Homer2 protein and mRNA (Figure 2A–F’). Notably, the apical pole and perinuclear region of odontoblasts and differentiating ameloblasts exhibited puncta enriched with Homer proteins. These cells also showed diffuse Homer2 and Homer3 immunostaining of the cytoplasm and plasma membranes (Figure 2D–F’). The vascular endothelium was enriched with Homer3 protein and transcripts and showed weak and moderate immunostaining for Homer1 and Homer2, respectively (Figure 2C’–F’).

At later postnatal stages, in both molars and incisors odontoblasts continue producing predentin and begin to form dentin matrix and ameloblasts differentiate into secretory ameloblasts producing enamel matrix. After completion of enamel secretion, secretory ameloblasts differentiate into maturation-stage ameloblasts, the cells responsible for enamel maturation into a hard tissue [51,55], and the papillary layer, an epithelial structure adjacent to maturation-stage ameloblasts, becomes morphologically evident. The papillary layer consists of cells of the stellate reticulum, stratum intermedium and outer dental epithelium and is penetrated by prominent vascular loops [56]. Other remarkable changes occurring at postnatal stages include molar root formation guided by a proliferating structure called Hertwig’s epithelial root sheath [50]. 

**Figure 3:**
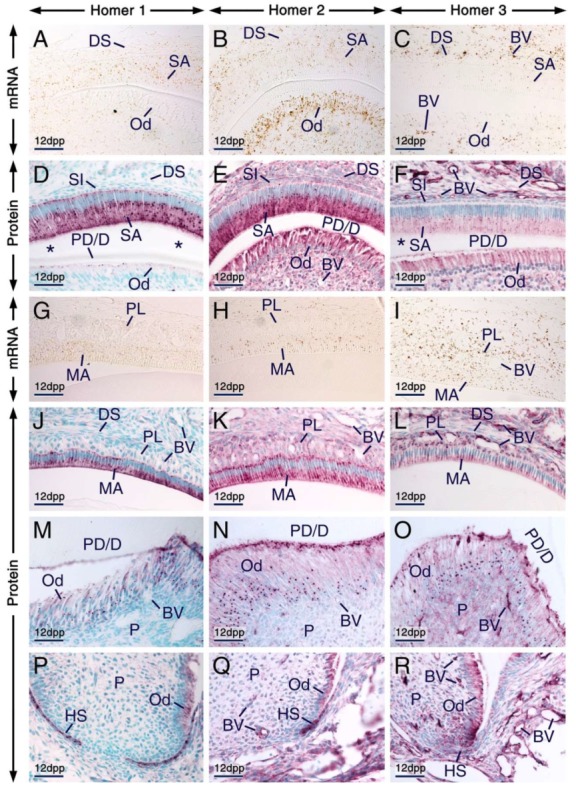
Expression patterns of Homer1, Homer2 and Homer3 during advanced stages of tooth formation. (**A**–**R**) Representative in situ hybridization (**A**–**C**,**G**–**I**) and immunohistochemistry (**D**–**F**,**J**–**R**) data showing the distribution patterns of *Homer1* (**A**,**G**), *Homer2* (**B**,**H**) and *Homer3* (**C**,**I**) transcripts (brown) and Homer1 (**D**,**J**,**M**,**P**), Homer2 (**E**,**K**,**N**,**Q**) and Homer3 (**F**,**L**,**O**,**R**) proteins (purple) in developing teeth at 12 days postpartum (12 dpp). (**A**–**F**) Sections across incisors at the level of the secretory stage of enamel formation. Secretory ameloblasts and young odontoblasts (cells facing a thin layer of predentin/dentin matrices) express the three Homer proteins and their encoding genes. The asterisks in (**D**,**F**) mark an artefactual space due to detachment of secretory ameloblasts from dentin. (**G**–**L**) Sections at the level of enamel maturation showing expression of the three Homer proteins and their encoding genes in maturation-stage ameloblasts and in the papillary layer. (**M**–**R**) Sections across molars showing expression of Homer proteins in mature odontoblasts (cells that have produced a thick layer of predentin/dentin) (**M**–**O**) and in Hertwig’s epithelial root sheath (**P**–**R**). Secretory ameloblasts, maturation-stage ameloblasts, odontoblasts and cells of the papillary layer exhibit puncta enriched with Homer proteins (**D**–**F**,**J**–**O**). Note that the Homer-positive(+) puncta in young odontoblasts (**D**–**F**) are less prominent than the Homer+ puncta in mature odontoblasts (**M**–**O**) and that the Homer3+ puncta in subsets of cells of the dental pulp are apparently smaller than the Homer3+ puncta in mature odontoblasts (**O**). Odontoblasts in the developing roots express Homer proteins (**P**–**R**). The endothelium of vascular loops penetrating the papillary layer (**I**,**L**) and of blood vessels in the dental pulp and dental sac mesenchyme (**C**,**F**,**I**,**L**,**O**,**R**) expresses Homer3 mRNA and protein and exhibits weak Homer1 (**D**,**J**,**M**,**P**) and moderate Homer2 (**E**,**K**,**N**,**Q**) immunostaining. BV, blood vessels; DS, dental sac; HS, Hertwig’s epithelial root sheath; MA, maturation-stage ameloblasts; Od, odontoblasts; P, dental pulp; PD/D, predentin/dentin matrices; PL, papillary layer; SA, secretory ameloblasts; SI, stratum intermedium. Scale bars: 50 µm (**A**–**R**).

We found that secretory ameloblasts, maturation-stage ameloblasts and cells of the papillary layer expressed Homer1, Homer2 and Homer3 proteins and their encoding genes (Figure 3A–L). In secretory ameloblasts and maturation-stage ameloblasts Homer proteins were not only detectable in cell membranes and/or in the cytoplasm but were also concentrated in puncta. The Homer1-positive(+), Homer2+ and Homer3+ puncta were enriched in the apical pole and were also detectable in the perinuclear region (Figure 3D–F,J–L). Cells of the papillary layer exhibited Homer1+ and Homer2+ puncta and were conspicuously enriched with Homer3+ puncta (Figure 3J–L).

All three Homer family members continued to be expressed in odontoblasts postnatally (Figure 3A–F,M–O). Both young odontoblasts, cells which produced predentin or the first layers of dentin in addition to predentin (predentin/dentin) (Figure 3D–F; see alsoFigure 2D’–F’), and relatively more mature odontoblasts which formed a thick layer of predentin/dentin (Figure 3M–O), exhibited Homer1+, Homer2+ and Homer3+ puncta in their apical pole and perinuclear region. Compared to the Homer2+ and Homer3+ puncta in young odontoblasts, the Homer2+ and Homer3+ puncta in mature odontoblasts were more conspicuous and apparently larger.

The endothelium of blood vessels penetrating the papillary layer, the dental sac mesenchyme and the dental pulp was enriched with Homer3 mRNA and protein and exhibited weak Homer1 and moderate Homer2 immunostaining (Figure 3A–R).

In developing roots, Hertwig’s epithelial root sheath was enriched with Homer1 and Homer2 proteins and exhibited weak Homer3 immunostaining (Figure 3P–R).

Taken together, these data show that the three Homer family members are expressed from early stages of odontogenesis onwards and displayed overlapping expression patterns in tooth-forming cells.

### 2.3. Expression Patterns of Homer1, Homer2 and Homer3 in the Developing Forebrain and Trigeminal Ganglion

In the E14.5 mouse brain, the expression patterns of *Homer1*, *Homer2* and *Homer3* transcripts detected by Digoxigenin-labelled riboprobes are accessible at Eurexpress.org [57]. Our in situ hybridization data obtained with oligonucleotide probes targeting *Homer1*, *Homer2* and *Homer3* in sections across the E14.5 mouse forebrain are consistent with data shown at Eurexpress.org, with the slight difference that oligonucleotide probes and the RNAscope technology used in this study generated strong hybridization signals and a higher signal to noise ratio compared to signals revealed by in situ hybridization with Digoxigenin-labelled riboprobes. 

**Figure 4:**
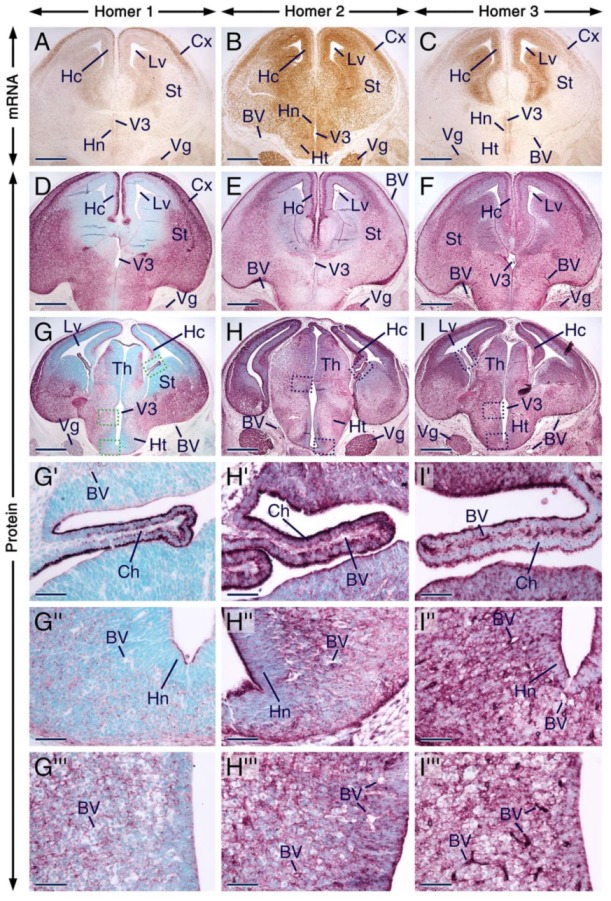
Expression patterns of Homer1, Homer2 and Homer3 in the developing forebrain and trigeminal ganglion. (**A**–**I**) Representative sections across the forebrain and trigeminal ganglion at embryonic day 14.5 (E14.5) after in situ hybridization revealing *Homer* transcripts (**A**–**C**) and immunostaining (**D**–**I**) for Homer proteins. Homer transcripts (brown) and Homer proteins (purple) are expressed in the trigeminal ganglion and in different regions of the forebrain, including the striatum, neocortex, thalamus, hypothalamus and in the hippocampal formation, and Homer proteins are enriched in differentiating fields and in the apical surface of the ventricular layer. (**G’**–**I’**) are magnified views of boxed areas in (**G**–**I**) showing that Homer1, Homer2 and Homer3 proteins are enriched in the apical surface of cells of the choroid plexus and that in these cells Homer1 and Homer3 are also concentrated in puncta. (**G’’**–**I’’**) are magnified views of boxed areas in (**G**–**I**) showing the distribution of Homer proteins in the anterior hypothalamic area and concentration of Homer1 and Homer2 in intracellular puncta. The three Homer proteins are also detectable in the apical surface of the hypothalamic neuroepithelium (**G’’**–**H’’**). (**G’’’**–**I’’’**) are magnified views of boxed areas in (**G**–**I**), showing that in other brain regions, the intracellular Homer-positive(+) puncta are less conspicuous than the Homer+ puncta in the anterior hypothalamic area. The endothelial lining of blood vessels in the brain and meninges expresses Homer3 transcripts and proteins (**C**,**F**,**I**–**I’’’**) and exhibits weak Homer1 (**D**,**G**–**G’’’**) and moderate Homer2 immunostaining (**E**,**H**–**H’’’**). BV, blood vessels; Ch, choroid plexus; Cx, neocortex; Hc, hippocampal formation; Ht, hypothalamus; Hn, hypothalamic neuroepithelium; Lv, lateral ventricle; St, striatum; Th, thalamus; Vg, trigeminal ganglion; V3, third ventricle. Scale bars: 500 µm (**A**–**I**) and 50 µm (**G’**–**I’’’**).

We found that *Homer1*, *Homer2* and *Homer3* mRNAs display overlapping expression patterns in the trigeminal ganglion as well as in various regions of the forebrain, including the hippocampal formation, neocortex, striatum, thalamus and hypothalamus (Figure 4A–C). The distribution of Homer proteins in the prenatal forebrain has hitherto not been investigated. In the postnatal brain, it has been shown that Homer transcripts accumulate in neuronal somata, whereas the proteins are enriched in postsynaptic densities, dendrites and axons [3,4,5,7,15,18], structures that are found in differentiated neurons. Although, overall, the distribution patterns of Homer proteins in the E14.5 forebrain matched the patterns of expression of their encoding transcripts, the proteins were concentrated in differentiating fields known to be rich in dendrites and axons, as well as in the apical surface of the ventricular layer (Figure 4A–I). The three Homer proteins were enriched in the neocortex, striatum, hippocampal formation, thalamus, hypothalamus and choroid plexus (Figure 4D–I’; Table S1). A closer look revealed interesting distribution patterns of Homer proteins in the choroid plexus and anterior hypothalamic area. The apical surface of cells of the choroid plexus was enriched with all three Homer proteins and these cells exhibited distinct perinuclear puncta enriched with Homer1 and Homer3 proteins (Figure 4G’–I’). In the anterior hypothalamus, Homer1 and Homer2 proteins were concentrated in perinuclear puncta but only a few puncta with Homer3 immunostaining were detectable in this region (Figure 4G’’–I’’). Homer3+ puncta in the anterior hypothalamus could, however, have been masked by the relatively strong Homer3 immunostaining. Cells in other brain regions also displayed Homer1+ and Homer2+ puncta but compared to the anterior hypothalamus, the puncta were less conspicuous (Figure 4G’’’–I’’’). The apical surface of the hypothalamic neuroepithelium was enriched with Homer1, Homer2 and Homer3 proteins (Figure 4G’’–I’’). All three Homer proteins were also expressed in the trigeminal ganglion (Figure 4G–I). 

Remarkably, the endothelial lining of blood vessels in the brain, meninges and choroid plexus was enriched with Homer3 protein and transcripts (Figure 4A–I’’’). By contrast, the vascular wall in these structures displayed weak immunostaining for Homer1. Homer2 immunostaining was moderate in the endothelium of blood vessels within the choroid plexus and meninges and weak in the endothelium of brain blood vessels (Figure 4D–I’’’). At 12 dpp, the vascular endothelium in brain and meninges expressed Homer3 protein and transcripts (Figure S1C,F) and showed weak and moderate immunostaining for Homer1 and Homer2, respectively (Figure S1D,E).

### 2.4. Expression Patterns of Homer1, Homer2 and Homer 3 in Other Developing Cephalic Regions 

#### 2.4.1. Eye, Olfactory and Respiratory Mucosae, Cranial Nerves, Salivary Glands, Rugae Palatinae, Palatal Medial Epithelial Seam and Skeletal Muscle 

**Figure 5:**
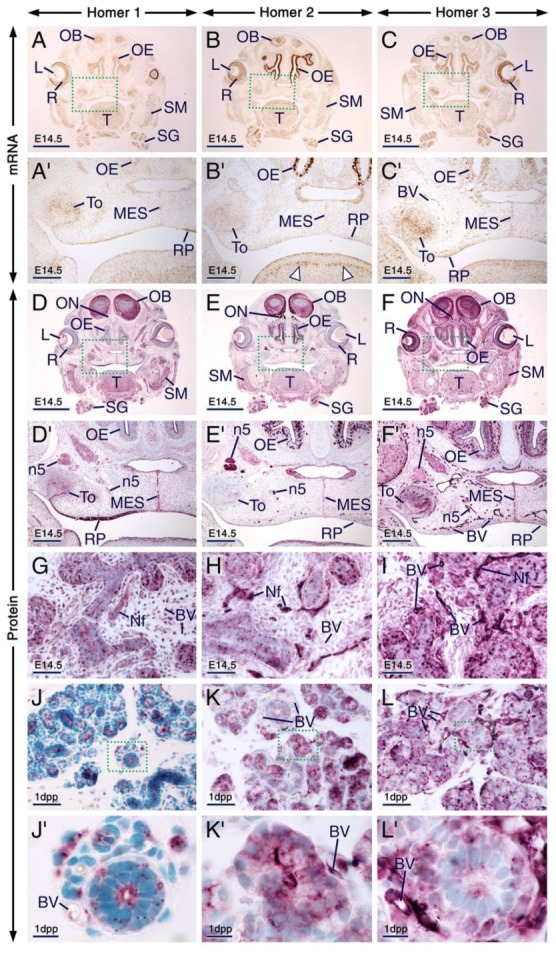
Expression patterns of Homer1, Homer2 and Homer3 in neuronal and non-neuronal cephalic tissues. (**A**–**I**) Representative sections across cephalic tissues at embryonic day 14.5 (E14.5) after in situ hybridization (**A**–**C**) and immunohistochemistry (**D**–**I**). (**A’**–**C’**) and (**D’**–**F’**) are magnified views of the boxed areas in (**A**–**C**) and (**D**–**F**), respectively. (**A**–**F’**) The *Homer1*, *Homer2* and *Homer3* mRNA expression patterns (brown) are consistent with the distribution patterns of their protein products (purple) in several cephalic structures, including the olfactory bulbs, retina, lens epithelium, olfactory epithelium, submandibular salivary glands, tooth, rugae palatinae, skeletal muscle and medial epithelial seam of the secondary palate. Note that *Homer2* mRNA is enriched in developing muscles of the tongue (arrowheads in **B’**). The olfactory epithelium displays strong Homer2 (**B**,**E**) but moderate Homer1 (**A**,**D**) and Homer3 (**C**,**F**) hybridization signals and immunolabelling. The olfactory and trigeminal nerves show moderate Homer1 (**D**,**D’**) and strong Homer2 (**E**,**E’**) immunostaining. Homer3 immunoreactivity is strong in olfactory nerves and moderate in trigeminal nerves (**F**,**F’**). (**G**–**I**) Homer1, Homer2 and Homer3 proteins are enriched in intracellular puncta in the epithelium of developing submandibular glands and the three Homer proteins are detectable in nerves within the glandular stroma. Note that vascular endothelium in cephalic tissues expresses Homer3 mRNA and protein and exhibits weak Homer1 and moderate Homer2 immunostaining. (**J**–**L**) Representative sections across submandibular salivary glands at 1 day postpartum (1 dpp) after immunostaining for Homer1 (**J**), Homer2 (**K**) and Homer3 (**L**). (**J’**–**L’**) are magnified views of the boxed areas in (**J**–**K**). In epithelial cells (acinar and tubular cells) of the glands Homer proteins show overlapping and distinct subcellular distribution patterns. Homer1 is enriched in the apical surface/membranes and in numerous intracellular puncta (**J**,**J’**); Homer2 is concentrated in the apical surface/membranes and is also detectable in the cytoplasm (**K**,**K’**); and Homer3 is detectable in the cytoplasm, cell membranes and puncta (**L**,**L’**). The Homer2-positive(+) and Homer3+ puncta are less conspicuous than the Homer1+ puncta in glandular epithelial cells (**J**–**L’**) and the Homer+ puncta in 1 dpp glands (**J**–**K**) are relatively smaller than the Homer+ puncta in E14.5 glands (**G**–**I**). BV, blood vessels; L, lens epithelium; MES, medial epithelial seam; Nf, nerve fibers (in salivary glands); n5, trigeminal nerve; OB, olfactory bulb; OE, olfactory epithelium; ON, olfactory nerve; R, retina; RP, rugae palatinae; SG, submandibular salivary glands; SM, skeletal muscle (masseter); T, tongue; To, tooth. Scale bars: 1mm (**A**–**F**), 200 µm (**A’**–**F’**), 50 µm (**G**–**L**) and 10 µm (**J’**–**L’**).

At E14.5, other developing cephalic tissues expressing the three Homer family members include the retina, lens epithelium, olfactory bulbs, olfactory epithelium, submandibular salivary glands, rugae palatinae, medial epithelial seam of the secondary palate and skeletal muscles (Figure 5A–I; Table S1). The distribution patterns of Homer proteins matched the expression patterns of their encoding transcripts (Figure 5), except that the transcripts were confined to neuronal somata, whereas the proteins were also detectable in cranial nerves, including the olfactory nerves and trigeminal nerves (Figure 5A–F’). 

Although the expression patterns of the three Homers overlapped, there were differences in the intensities of hybridization signals and immunolabelling. For example, the olfactory epithelium was enriched with *Homer2* mRNA and Homer2 protein (*Homer2*/Homer2), rugae palatinae were enriched with *Homer1*/Homer1 and the retina as well as the vascular endothelium were enriched with *Homer3*/Homer3 (Figure 5A–F’). Cranial nerves also exhibited different intensities of Homer immunostaining: The olfactory and trigeminal nerves showed moderate Homer1 (Figure 5D,D’) and strong Homer2 (Figure 5E,E’) immunostaining, however these nerves displayed strong (olfactory nerves) and moderate (trigeminal nerves) Homer3 immunostaining (Figure 5F,F’). *Homer1*/Homer1, *Homer2*/Homer2 and *Homer3*/Homer3 were also expressed in skeletal muscles, including the masseter muscle and developing muscles of the tongue (Figure 5A–F). The finding of expression of the three Homer family members in skeletal muscle is concordant with previous data generated by RT-qPCR analyses [29]. In the developing tongue, relatively strong *Homer2* hybridization signals and Homer2 immunolabelling were confined to a region that likely comprises differentiating muscle cells (Figure 5B’). Accordingly, previous studies have shown that *Homer2* expression is transiently upregulated at E14.5 in mouse embryos and that Homer2 plays a critical role in myoblast differentiation [58]. 

All three Homer family members were expressed in submandibular salivary glands at E14.5 and at 1 dpp (Figure 5A–L; Table S1). At E14.5, the three Homer proteins were detectable in nerves innervating salivary glands (Figure 5G–I). In the epithelium of the embryonic submandibular glands the three Homer proteins were concentrated in puncta and were also detectable in cell membranes (Figure 5G–I). In postnatal submandibular glands, Homer2 and Homer3 proteins were detectable in the cytoplasm and cell membranes of acinar and duct cells but in these cells Homer1 protein was concentrated in the apical surface as well as in puncta (Figure 5J–L’). Remarkably, the Homer+ puncta in the embryonic glands appeared relatively larger than the Homer1+ puncta in the postnatal glands (Figure 5G–L). In the embryonic and postnatal submandibular glands the vascular endothelium exhibited weak Homer1, moderate Homer2 and strong Homer3 immunostaining (Figure 5G–L’). 

**Figure 6:**
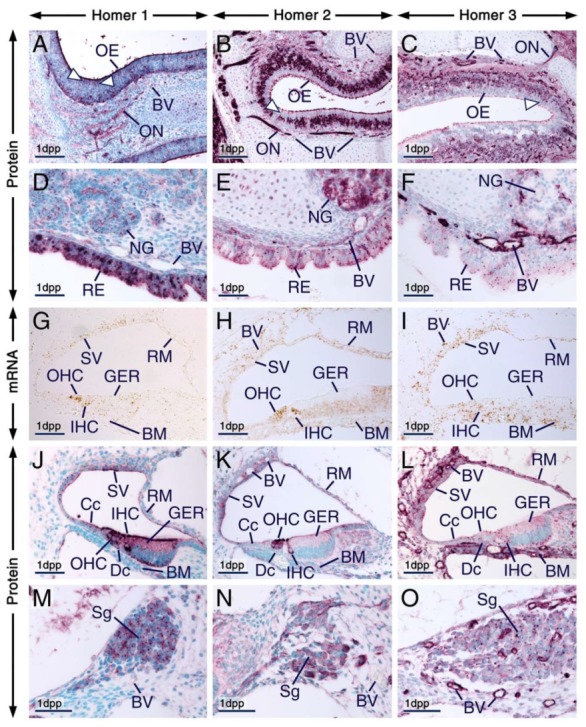
Expression patterns of Homer1, Homer2 and Homer3 in the olfactory and respiratory mucosae and in the cochlea. (**A**–**O**) Representative immunohistochemistry (**A**–**F**,**J**–**O**) and in situ hybridization (**G**–**I**) data in sections across the nasal cavity (**A**–**F**) and cochlea (**G**–**O**) at 1 day postpartum (1 dpp) showing the distribution patterns of Homer proteins (purple) and their encoding genes (brown). (**A**–**C**) Homer1 (**A**), Homer2 (**B**) and Homer3 (**C**) proteins are enriched in the apical surface (likely cilia of olfactory neurons and microvilli of supporting cells) of cells of the olfactory epithelium and are also detectable in intracellular puncta. Dendrites (arrowheads in **A**–**C**) and axons, the latter forming the olfactory nerves, are Homer1-positive(+), Homer2+ and Homer3+, indicating that some of the Homer+ cells in the olfactory epithelium are olfactory neurons. (**D**–**F**) The respiratory epithelium and nasal glands express Homer1 (**D**), Homer2 (**E**) and Homer3 (**F**) proteins. In cells of the respiratory epithelium the Homer1+ puncta are more prominent than the Homer2+ and Homer3+ puncta. In nasal gland acinar cells Homer1 is enriched in the apical membrane and in puncta, Homer2 is readily detectable in cell membranes and in the cytoplasm and Homer3+ puncta are detectable in subsets of acinar cells. Note that the Homer1+ puncta in nasal gland acinar cells are small compared to the Homer1+ puncta in cells of the respiratory epithelium (**D**). The endothelial lining of blood vessels in the olfactory (**A**–**C**) and respiratory (**D**–**F**) mucosae shows weak Homer1, moderate Homer2 and strong Homer3 immunolabelling. (**G**–**O**) Expression patterns of *Homer* transcripts (**G**–**I**) and their protein products (**J**–**O**) in the cochlea. *Homer1* (**G**), *Homer2* (**H**) and *Homer3* (**I**) mRNAs are detectable in various cells of the developing organ of Corti, including inner (IHC) and outer (OHC) hair cells as well as in cells forming the stria vascularis, basilar membrane and Reissner’s membrane (**G**–**I**). *Homer1* and *Homer2* transcripts are enriched in IHC and OHC (**G**,**H**). Homer1 (**J**), Homer2 (**K**) and Homer3 (**L**) proteins show overlapping but also distinct subcellular localization in cells of the organ of Corti. In IHC and OHC Homer1 and Homer2 are enriched in the apical surface (likely in stereocilia) and in perinuclear puncta and are also detectable in the cytoplasm. By contrast, Homer3 protein is detectable in puncta at the apical surface of IHC and OHC and in perinuclear puncta in IHC. Other cells of the organ of Corti, including cells of the greater epithelial ridge (GER), cells of Claudius and Deiter’s cells, exhibit Homer1+ and Homer3+ perinuclear puncta and show moderate Homer1, very weak Homer2 and weak Homer3 immunostaining in the cytoplasm. Homer1 is also enriched in the apical (endolymphatic) surface of the GER. Cells of the stria vascularis and Reissner’s membrane exhibit Homer1+ and Homer3+ puncta and cytoplasmic Homer2 immunostaining. Note that in IHC and OHC the Homer1+ puncta are large as compared to the Homer2+ and Homer3+ puncta and that the Homer1+ puncta in IHC and OHC are large compared to the Homer1+ puncta in the cells of Claudius and in cells of the GER and stria vascularis. (**M**–**O**) Neurons of the cochlear spiral ganglion show diffuse cytoplasmic immunostaining for Homer1 (**M**), Homer2 (**N**) and Homer3 (**O**) and are enriched with Homer1+ and Homer3+ puncta. Note that the vascular endothelium in the cochlea exhibits weak Homer1, moderate Homer2 and strong Homer3 immunolabelling (**J**–**O**). BM, basilar membrane; BV, blood vessels; Cc, cells of Claudius; Dc, Deiter’s cells; GER, greater epithelial ridge; IHC, inner hair cells; NG, nasal glands; OE, olfactory epithelium; ON, olfactory nerve; RE, respiratory epithelium; RM, Reissner’s membrane; Sg, spiral ganglion; SV, stria vascularis. Scale bars: 100 µm (**A**–**C**) and 50 µm (**D**–**O**).

At 1 dpp, Homer proteins continued to be detectable in the olfactory epithelium and olfactory nerves and cells of the olfactory epithelium exhibited puncta enriched with Homer proteins (Figure 6A–C; Table S1). Homer2 immunostaining of the olfactory epithelium and olfactory nerves is in agreement with previous findings [18]. The apical surface of the olfactory epithelium, known to consist of microvilli of supporting cells and cilia of olfactory sensory neurons [59], exhibited strong immunostaining for Homer1 and Homer2 and relatively moderate Homer3 immunostaining (Figure 6A–C). The endothelial lining of blood vessels within the lamina propria of the olfactory mucosa showed strong anti-Homer3, moderate anti-Homer2 and weak anti-Homer1 immunoreactivities (Figure 6A–C). 

The respiratory epithelium of the nasal cavity as well as nasal glands also expressed Homer proteins (Figure 6D–F; Table S1). Remarkably, cells of the respiratory epithelium displayed puncta enriched with Homer1, Homer2 and Homer3 proteins and the Homer1+ puncta were relatively large (Figure 6D–F). Homer1 and Homer2 proteins were readily detectable in nasal gland acinar cells and displayed overlapping as well as distinct subcellular localizations. Both proteins were enriched in the apical membrane of acinar cells. However, whereas Homer1 was also enriched in perinuclear puncta, Homer2 was detectable in the cytoplasm as well (Figure 6D,E). By contrast, Homer3 was detectable in puncta in subsets of nasal gland acinar cells (Figure 6F). In the lamina propria of the respiratory mucosa the vascular endothelium displayed weak Homer1, moderate Homer2 and strong Homer3 immunolabelling (Figure 6D–F).

#### 2.4.2. Cochlea

In situ hybridization in sections across the cochlea at 1dpp revealed expression of *Homer1*, *Homer2* and *Homer3* transcripts in the organ of Corti, stria vascularis and Reissner’s membrane. While hybridization signals for *Homer3* were evenly distributed in the organ of Corti, the strongest *Homer1* and *Homer2* hybridization signals were detectable in inner hair cells (IHC) and outer hair cells (OHC) of the organ of Corti (Figure 6G–I). *Homer3* mRNA was also expressed in the vascular endothelium and in the basilar membrane (Figure 6I). At 1 dpp, the basilar membrane comprises a vascularized mesenchyme [60]. All three Homer proteins were expressed in the organ of Corti and adjacent tissues of the cochlea (Figure 6J–O; Table S1). In IHC and OHC Homer1 immunostaining was detectable in the cytoplasm and was concentrated in the apical surface (likely hair cell stereocilia) as well as in perinuclear puncta. In addition, in the organ of Corti Homer1 protein was readily detectable in cells of the greater epithelial ridge (GER) and was enriched in their apical (endolymphatic) surface; the tectorial membrane covering the GER was devoid of Homer1 immunostaining. Homer1 protein was also concentrated in perinuclear puncta in cells of the GER as well as in the cells of Claudius, Deiter’s cells and in cells of the stria vascularis (Figure 6J).

It has been shown previously that Homer2 protein localizes at stereocilia of IHC and OHC [35]. Accordingly, we detected robust Homer2 immunostaining in stereocilia of IHC and OHC. However, we also detected Homer2+ perinuclear puncta and cytoplasmic Homer2 immunostaining in IHC and OHC (Figure 6K). By contrast Homer2 immunostaining in the GER, Deiter’s cells and in cells of Claudius was weak (Figure 6K). Remarkably, in IHC and OHC, the Homer2+ perinuclear puncta appeared relatively smaller than the Homer1+ perinuclear puncta (Figure 6J,K). Homer3 immunostaining was strong in the basilar membrane and Homer3+ puncta were readily detectable in the apical surface of IHC and OHC as well as in the perinuclear region of IHC, basal cells of the GER, cells of Claudius, Deiter’s cells and in subsets of cells within the stria vascularis and Reissner’s membrane (Figure 6L). The vascular endothelium in the cochlea showed weak, moderate and strong immunostaining for Homer1, Homer2 and Homer3, respectively (Figure 6J–L). In the spiral ganglion, all three Homer family members were readily detectable and were enriched in intracellular puncta; however, the Homer1+ and Homer3+ puncta were more conspicuous than the Homer2+ puncta (Figure 6M–O; Table S1).

#### 2.4.3. Tongue, Taste Buds and Bone 

**Figure 7:**
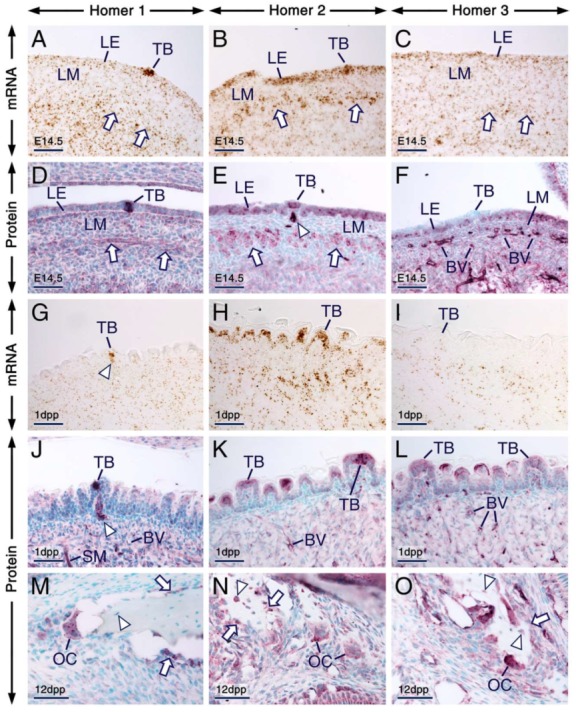
Expression patterns of Homer1, Homer2 and Homer3 in the tongue and alveolar bone. (**A**–**O**) Representative in situ hybridization (**A**–**C**,**G**–**I**) and immunohistochemistry (**D**–**F**,**J**–**O**) data showing the distribution patterns of *Homer* transcripts (brown) and Homer proteins (purple). (**A**–**F**) Sections across the developing tongue at embryonic day 14.5 (E14.5). *Homer1* mRNA (**A**) and Homer1 protein (**D**) are enriched in developing taste buds and are detectable in the lingual epithelium (LE), developing muscles of the tongue (arrows) and in the lingual mesenchyme (LM). *Homer2* mRNA (**B**) and Homer2 protein (**E**) are expressed in the LE, including in developing taste buds and are enriched in developing muscle of the tongue (arrows). Homer2 protein is also detectable in nerves innervating developing fungiform papillae (arrowhead in **E**). *Homer3* mRNA (**C**) and Homer3 protein (**F**) are expressed in the LE, LM and in the vascular endothelium, whereas developing taste buds are virtually devoid of Homer3 immunostaining. (**G**–**L**) Sections across the tongue at 1 day postpartum (1 dpp). *Homer1* mRNA (**G**) and Homer1 protein (**J**) are enriched in nerves innervating fungiform papillae (arrowheads in **G**,**J**) and in taste buds. The LE and taste buds express *Homer2* mRNA (**H**) and Homer2 protein (**K**) and exhibit weak hybridization signals (**I**) and immunostaining **(L**) for Homer3. By contrast, *Homer3* transcripts and Homer3 protein are readily detectable in the vascular endothelium. (**M**–**O**) sections across the alveolar bone at 12 dpp. Homer1 (**M**), Homer2 (**N**) and Homer3 (**O**) proteins are expressed in osteoblasts (arrows in **M**,**N**,**O**) and osteoclasts and Homer2 and Homer3 but not Homer1, are also detectable in osteocytes (arrowheads in **M**,**N**,**O**). Note that in bone cells Homer proteins are enriched in puncta. BV, blood vessels; LE, lingual epithelium; LM, lingual mesenchyme; OC, osteoclast; TB, taste bud; SM, muscles of the tongue. Scale bars: 50 µm (**A**–**O**).

At E14.5 and at 1 dpp, Homer1, Homer2 and Homer3 transcripts and/or proteins were expressed in the developing tongue (Figure 7; Table S1; see also Figure 5). In a previous study, we showed that Homer1 protein is expressed in taste buds of postnatal mice [61]. In the present study, we found that not only postnatal taste buds (Figure 7G,J) but also embryonic differentiating taste buds (Figure 7A,D) expressed Homer1 protein and mRNA. At 1 dpp, Homer1 protein and its encoding gene were also expressed in cells within nerves innervating fungiform papillae (Figure 7G,J). At E14.5 (Figure 7B,E) and 1 dpp (Figure 7H,K), Homer2 mRNA and protein were readily detectable in the entire lingual epithelium, including in taste buds. At E14.5, although Homer3 protein and mRNA were detectable in the lingual epithelium (Figure 7C,F), differentiating taste buds were devoid of Homer3 immunostaining (Figure 7F). By 1 dpp, taste buds showed weak *Homer3* hybridization signals and weak Homer3 immunostaining (Figure 7I,L).

The vascular endothelium in the tongue showed weak, moderate and strong immunostaining for Homer1, Homer2 and Homer3, respectively (Figure 7D–F,J–L). 

In prenatal and postnatal mice, osteocytes showed immunostaining for Homer2 and Homer3 and were devoid of Homer1 immunolabelling, whereas osteoblasts displayed immunostaining for the three Homer proteins (Figure 7M–O; Table S1; see also Figure 2D–F). Furthermore, all three Homer proteins were readily detectable in osteoclasts and were enriched in puncta (Figure 7M–O; Table S1).

## 3. Discussion

The present study provided several insights: (1) In general, all three Homer family members are expressed ubiquitously in cephalic tissues. (2) In developing organs, Homer1, Homer2 and Homer3 are expressed in undifferentiated/proliferating cells, differentiating cells as well as in differentiated cells. (3) Generally, in a given cephalic structure, Homer1, Homer2 and Homer3 exhibit overlapping expression patterns. (4) The three Homer proteins display similar but also distinct subcellular localizations in several cells types. (5) In a multitude of cell types, Homer proteins are enriched in puncta. (6) The endothelial lining of blood vessels, including brain blood vessels, is enriched with Homer3. 

### 3.1. The Three Homer Family Members are Expressed Ubiquitously in Developing Cephalic Tissues 

Ca^2+^ signaling is a fundamental mechanism involved in a multitude of cellular processes, including proliferation, differentiation, apoptosis, gene transcription and specialized cell functions such as neuronal activity, secretion, contraction [62] and taste perception [63]. Homer proteins are known to play key roles in various types of Ca^2+^ signaling by directly or indirectly modulating the activities of Ca^2+^-handling proteins in a wide variety of cell types, including neurons [7,8,13,14,37,38,64], acinar cells of the pancreas [32] and parotid glands [33], platelets [39], striated [34,58] and smooth [40] muscle, T cells [65], osteoblasts [66] and osteoclasts [67]. 

Homer proteins have also been shown to interact with F-actin [5,68], an essential cytoskeletal component with key cellular functions such as regulation of cell shape and polarity, cell division and adhesion [69], cell migration [70], intracellular transport [71], endocytosis [72] and exocytosis [73]. Furthermore, Homer proteins have been reported to directly interact with regulators of cytoskeletal actin, including the protocadherin FAT1 [74], Drebrin [75,76] and the Cdc42 small GTPase [75]. 

In light of these observations, the ubiquity of the expression of the three Homer family members is not surprising. Besides being expressed during embryonic development in proliferating and differentiating cells in various developing cephalic tissues and organs, including the tooth, hippocampal formation, tongue and submandibular glands, the three Homer family members were also expressed in differentiated cells/tissues with specialized functions. The latter category includes cells and tissues with secretory functions such as ameloblasts, odontoblasts, osteoclasts, glandular epithelia and the choroid plexus, cells involved in reabsorption of extracellular components such as maturation-stage ameloblasts and osteoclasts, as well as cells involved in gustatory (taste), auditory and olfactory perceptions. These observations suggest that Homer proteins have multifaceted biological functions.

In this study we revealed that Homer2 and Homer3 are expressed in osteoblasts and confirmed previous findings of Homer1 expression in osteoblasts [66]. While the role of Homer2 and Homer3 in these bone cells is at present unknown, it has been shown that in response to extracellular Ca^2+^, Homer1 forms a complex with the Calcium sensing receptor and mTOR complex 2, leading to activation of the Serine/Threonine kinase AKT, thereby inhibiting apoptosis and promoting AKT-dependent β-catenin stabilization and cell differentiation [66]. Furthermore, *HOMER1* has been identified among other genes as playing a role in female osteoporosis [77].

To our knowledge, whether osteoclasts express Homer proteins has not been investigated. Here we show that osteoclasts produce all three Homer proteins, suggesting that Homer family members have overlapping functions in these cells. Indeed, it has been shown that mutant mice lacking the functions of both *Homer2* and *Homer3* (*Homer2/Homer3* DKO) exhibit decreased bone density and mechanistic studies in vitro suggest that the phenotype of *Homer2/Homer3* DKO mice is likely caused by increased osteoclastogenesis and bone metabolism since Homer2 and Homer3 were found to regulate RANKL-induced osteoclast differentiation through modulation of NFATc1 activity [67]. As osteoblasts express Homer2 and Homer3 proteins (this study), the bone anomalies in *Homer2/Homer3* DKO mice could also be caused by defective osteoblast function.

Among the short Homer proteins, only the short Homer1 splice isoforms, namely Homer1a and Ania-3, have been characterized in terms of expression and function [7,78,79]. Unlike the long Homer isoforms, which are constitutively expressed in both neuronal and non-neuronal tissues [3,6,7,28,29,30,31,32,33,78,79], Homer1a and Ania-3 have been shown to be neuron-specific splice isoforms [1,2,78] and to be rapidly and transiently upregulated upon neuronal stimulation [1,2,3,6,78,79]. We found that at E14.5, in addition to other brain regions, the hippocampal formation was enriched with Homer1 mRNA and protein. In the rat *Homer1a/vesl* expression levels have been shown to be developmentally regulated, being low in embryos at E20 as well as in adults and high during neuronal differentiation and synaptogenesis, which occur during postnatal weeks 2–3 [2]. These findings suggest that Homer1a and Ania3 may not be produced in non-neuronal cephalic tissues and that in the prenatal hippocampal formation the short Homer1 isoforms may not be predominant. 

### 3.2. Probable Roles for Homer Proteins During Tooth Formation 

In the developing tooth, the three Homer family members were expressed from initiation of tooth formation onwards, suggesting roles for these proteins in proliferation, differentiation and function of tooth-forming cells. However, no tooth phenotype has been reported in mice lacking the function of *Homer1*, *Homer2* or *Homer3*. While tooth anomalies might have been overlooked in *Homer*-deficient mice, the lack of a tooth phenotype might be due to functional redundancy between the three Homer proteins, since tooth-forming cells exhibited overlapping expression patterns of the three Homer family members. 

While delineation of the precise role of Homer proteins during tooth formation awaits further studies, several observations suggest that Homer proteins may be involved in Ca^2+^ transport and Ca^2+^ signaling in ameloblasts, stratum intermedium and in the papillary layer: (1) Our study showed that secretory ameloblasts, maturation-stage ameloblasts, the stratum intermedium and the papillary layer produce the three Homer proteins. (2) Ameloblasts are endowed with an elaborate system for Ca^2+^ transport and Ca^2+^ signaling, enabling them to maintain their structural integrity and ensure proper enamel formation and cells of the stratum intermedium and papillary layer also express proteins involved in Ca^2+^ dynamics [42,45,51]. Indeed, ameloblasts express several proteins involved in Ca^2+^ signaling, including Orai1 and Stim1 [43,44,45,46,47] as well as IP3Rs, the latter being also expressed in the stratum intermedium and papillary layer [44]. Furthermore, it has been shown that the stratum intermedium and papillary layer express PMCA1 and that ameloblasts express both PMCA1 and PMCA4 [42]. (3) Previous studies have shown that Homer proteins bind to IP3Rs [7,12], that Homer1 interacts directly with Stim1 and indirectly with Orai1 in a Ca^2+^-dependent way during agonist-induced SOCE activation in human platelets [39] and that in rat balloon-injured arteries Homer1 binds Orai1 channels and is required for SOCE in vascular smooth muscle cells [40]. (4) Finally, Homer proteins have been shown to bind PMCAs [33,37,80] and to modulate PMCA-mediated Ca^2+^-clearance in rat hippocampal neurons [38] and in mouse salivary gland acinar cells [33]. 

We found that odontoblasts express all three Homer family members. In odontoblasts Ca^2+^ signaling and Ca^2+^ transport mechanism are involved in sensory transduction upon exposure of dentin to diverse stimuli and in dentin mineralization [81,82,83,84,85,86,87,88,89,90]. It has been shown that odontoblasts express Orai1 [45,91] as well as Stim1 and Stim2 [49] and that SOCE is activated upon intracellular Ca^2+^ store depletion in odontoblasts [85,90]. Ca^2+^ influx into odontoblasts is also mediated by voltage-gated L-type channels [81,83,92] and odontoblasts express L-type Ca_v_1.2 channels [93]. Interestingly, Stim1 protein has been shown to interact with Ca_v_1.2 channels and to inhibit their gating activity [94]. Furthermore, Homer proteins have been shown to associate with both Stim1 and Ca_v_1.2 channels in response to Ca^2+^ store depletion but not under resting conditions [95]. These findings together with our data prompt the question of whether Homer proteins are involved in modulating Ca^2+^ transport and Ca^2+^ signaling in odontoblasts.

### 3.3. Overlapping Expression Patterns and Distinct Subcellular Localization of Homer Proteins

In general, the three Homer family members showed overlapping expression patterns in a given cephalic tissue (Table S1). However, in several instances the intensities of Homer hybridization signals and/or immunostaining were different. Examples illustrating this phenomenon include cochlear hair cells in which among the three Homer family members, Homer1 and Homer2 are predominant and the vascular endothelium in which Homer3 is the most prominent family member. 

Furthermore, our study revealed that in several cell types Homer1, Homer2 and Homer3 proteins exhibit similar as well as different subcellular localizations, suggesting that the proteins may exert redundant and/or distinct functions in a given cell type. That Homer proteins can have redundant roles is illustrated by findings showing that Homer2 and Homer3 are both involved in T-cell activation through modulation of NFATc-dependent signaling and that the impact of loss of Homer3 on T cell activation can be prevented by Homer2 [65].

Cell types in which the three Homer proteins showed overlapping subcellular localizations include ameloblasts, cells of the olfactory epithelium, as well as epithelial cells of the choroid plexus, where the three Homer proteins were enriched in the apical membrane and/or in the apical pole.

Homer proteins do not contain membrane-spanning or highly hydrophobic domains [3]. Therefore, the localization of Homer proteins in the plasma membrane is likely mediated by their interaction with membrane-associated proteins.

Overlapping but also differential subcellular distribution of Homer proteins was also observed in several cell types, including cells of the organ of Corti and nasal gland acinar cells. Indeed, both Homer1 and Homer2 were enriched in the apical membrane of nasal gland acinar cells. However, Homer2 was also detectable in the cytoplasm, whereas Homer1 was in addition concentrated in perinuclear puncta.

Previous studies have shown that Homer2 is enriched in stereocilia of inner (IHC) and outer (OHC) hair cells of the organ of Corti [35]. Our study revealed that all three Homer family members and their encoding genes were expressed in the organ of Corti, including in IHC and OHC and that cells of the organ of Corti display similar and distinct subcellular localizations of Homer proteins (Table S1). Homer2 was readily detectable not only in the apical surface of IHC and OHC, which likely represents Homer2 protein in stereocilia but also in perinuclear puncta. In IHC and OHC Homer1 protein was enriched in the apical surface and in perinuclear puncta, whereas Homer3 protein was concentrated in puncta localized at the apical surface of IHC and OHC as well as in perinuclear puncta within IHC. The overlapping expression patterns of Homer1 and Homer2 in IHC and OHC suggest that Homer1 and Homer2 may exert compensatory functions to offset loss-of-function of one family member. However, patients with a *HOMER2* p.Arg185Pro mutation in the coiled-coil domain-encoding region exhibit impaired hearing and *Homer2* loss-of-function in mice causes progressive hearing loss [35], demonstrating that Homer2 has a non-redundant function which is critical for auditory acuity. 

Besides the organ of Corti, we found that the three Homer family members were also expressed in the spiral ganglion and in cells forming the stria vascularis and Reissner’s membrane. Homer3 has been shown to directly interact with WW domain-binding protein 2 (WBP2) in glioma cells [96]. Interestingly, it has been demonstrated that WBP2 is expressed in several cochlear structures, including the organ of Corti, Reissner’s membrane, stria vascularis and the spiral ganglion, that WBP2 function is crucial for normal hearing in humans and in mice and that hearing impairment in *Wbp2*-deficient mice is caused by defects in afferent nerve terminals underneath IHC and OHC combined with structural and molecular anomalies of ribbon synapses [97]. Loss-of-function of *Homer2* in mice has no negative impact upon the cytological structure of IHC and OHC [35]. These observations together with our findings raise the question of whether loss of more than one Homer family member would cause abnormal development of cochlear structures, including the organ of Corti, stria vascularis, Reissner’s membrane and spiral ganglion.

### 3.4. Homer Proteins Are Enriched in Puncta

A salient and frequent phenomenon revealed in this study is the punctate distribution pattern of the three Homer proteins in various cell types (Table S1). Furthermore, the Homer+ puncta localize at various subcellular regions: the apical pole and/or perinuclear region of cells. In polarized cells, such as ameloblasts and cells of the nasal respiratory epithelium, the Homer+ puncta were enriched in the apical pole of the cells. Homer+ puncta were also readily detectable in the perinuclear region of both polarized and non-polarized cells in various cephalic structures, including developing teeth, the choroid plexus, the organ of Corti, nasal glands, salivary glands and nasal respiratory epithelium. 

In some instances, Homer+ puncta were of different sizes: In the respiratory epithelium, the Homer1+ puncta were apparently larger than the Homer2+ and Homer3+ puncta; in mature odontoblasts the Homer2+ and Homer3+ puncta appeared larger than the Homer+ puncta in young odontoblasts; in embryonic submandibular salivary glands the Homer+ puncta were to a large extent larger than the Homer1+ puncta in postnatal submandibular glands; and in IHC and OHC of the cochlea the Homer1+ puncta were apparently larger than the Homer2+ and Homer3+ puncta. It is unlikely that the apparent different sizes of Homer+ puncta is an artefact, since other cells within the same tissues exhibiting cells with large Homer+ puncta or cells in adjacent tissues displayed relatively small Homer+ puncta. For example the Homer1+ puncta in nasal gland acinar cells were less prominent than the Homer1+ puncta in cells of the respiratory epithelium; the Homer3+ puncta in subsets of dental pulp mesenchymal cells were relatively smaller than the Homer3+ puncta in odontoblasts; in the epithelium of embryonic submandibular glands, subsets of cells with small Homer+ puncta were interspaced with cells exhibiting large Homer+ puncta; and in the organ of Corti the Homer1+ puncta in cells of the GER were apparently smaller than the Homer1+ puncta in IHC and OHC. It is thus likely that differences in the amounts of Homer proteins at puncta may account for the detection of Homer+ puncta of different sizes.

To our knowledge, this is the first report showing the localization of Homer proteins in puncta in a wide range of non-excitable cells in vivo. Indeed, hitherto only neurons and skeletal muscle have been shown to exhibit a punctate pattern of Homer protein distribution. In neurons Homer+ puncta localize at postsynaptic densities [3,5,15,16,17,18] and in human skeletal muscle Homer+ puncta localize at the subsynaptic domain of neuromuscular junctions [31].

Biochemical and structural studies showed that Homer proteins form tetramers [16,17] and multimers [15] via their coiled-coil domain and that as a whole the proteins form a dumbbell-like structure with two EVH1 domains at each end, a configuration that enables Homer proteins to bind four different target proteins [16,17]. Furthermore, Homer multimerization/tetramerization is critical for the subcellular localization of Homer proteins and clustering of Homer-binding partners [15,16,17] and several studies indicate that proper subcellular localization of Homer proteins requires their association with Homer-binding proteins [5,65,98,99].

In light of these findings, it is likely that the Homer+ puncta observed in this study may represent Homer tetramers or multimers complexed with Homer-binding proteins within cellular microdomains.

The central nervous system and other tissues and cells such as cochlear hair cells and the olfactory epithelium, exhibited strong Homer hybridization signals and immunostaining. However, other cell types and tissues exhibited weak Homer hybridization signals, yet they showed robust Homer immunostaining, including in puncta. While this may be due to the different methods used to detect Homer mRNAs and Homer proteins, it could also be the result of differences in the stability of mRNAs and proteins. Indeed, it has been shown that tetramerization of Homer proteins contributes to a slow turnover rate [16].

### 3.5. The Vascular Endothelium Is Enriched with Homer3 and also Produces Homer1 and Homer2

To our knowledge, our study is the first to report that the endothelial lining of blood vessels in cephalic structures, including the brain, is enriched with Homer3 protein and mRNA and shows weak Homer1 and moderate Homer2 immunostaining. The vascular endothelium is involved in angiogenesis, a process during which new blood vessels develop from preexisting ones and plays a role in sensing chemical and physical changes in blood [100]. Whether Homer proteins, particularly Homer3 and Homer2, play any role in the biology of endothelial cells remains an open question. It is noteworthy that endothelial cells respond to pro-angiogenic signals by increasing intracellular Ca^2+^ concentration as a result of activation of the SOCE mechanism and other Ca^2+^ channels, including TRPC channels [100]. Besides directly interacting with key proteins of SOCE [39,40,95], Homer proteins also bind and modulate the activity of TRPC channels [11,101,102]. Other binding targets of Homer proteins include Drebrin [75,76], an F-actin-binding protein with a key role in maintenance of endothelial integrity [103]. These observations suggest that Homer proteins may be involved in controlling endothelial cell function and structural integrity.

## 4. Materials and Methods 

### 4.1. Ethics Statement 

All the procedures that involve the use of mice were reviewed and approved by the Animal Research Ethics Committee in Göteborg (Dnr. 174-2013 (12 November 2013) and Dnr. 5.8.18-15468/2018 (5 December 2018)).

### 4.2. Tissue Processing, Immunohistochemistry and in Situ Hybridization

For immunohistochemistry and in situ hybridization, cephalic tissues from mouse embryos (*n* = 2 at E12.5, *n* = 3 at E13.5 and *n* = 3 at E14.5) and postnatal mice (*n* = 3 at 1 dpp and *n* = 3 at 12 dpp) were fixed in 4% paraformaldehyde in phosphate buffered saline (PBS) and processed for paraffin embedding as described previously [104].

The affinity-purified rabbit anti-Homer1 (product No. 12433-1-AP) was obtained from Proteintech Group (Manchester, UK). The affinity-purified rabbit anti-Homer2 (product No. NBP1-85487) and the affinity-purified rabbit anti-Homer3 (product No. NBP2-32607) were from Novus Biologicals (Abingdon, UK). Immunohistochemistry was carried out in dewaxed tissue sections as described previously [61,104,105]. Briefly, following antigen unmasking in 10 mM citrate buffer, pH 6, endogenous peroxidase was blocked by incubating the sections in 3% hydrogen peroxide in methanol. Thereafter, non-specific binding sites were blocked for 1 h with PBS containing 5% normal goat serum, 0.1% bovine serum albumin (BSA) and 0.1% Triton-X-100. Subsequently, the sections (test sections) were incubated overnight at 4 °C with anti-Homer1, anti-Homer2 or anti-Homer3 diluted 1:2000, 1:1000 and 1:3000, respectively, in PBS/BSA/Triton-X-100. Visualization of the distribution of Homer proteins was carried out using the biotinylated tyramide amplification system as described previously [104,105] and the sections were counterstained with methyl green which leads to green/blue staining of cellular nuclei. For assessment of antibody specificities, tissue sections (negative controls) were processed under the same conditions as those for the test sections, except that the primary antibodies were omitted.

For detection of *Homer1*, *Homer2* and *Homer3* transcripts by in situ hybridization on dewaxed sections, we used oligonucleotide probes and the Advanced Cell Diagnostics RNAscope technology (Bio-Techne, Oxon, UK). The probes were as follows: Mm-*Homer1* (gene ID: 26556; target sequence: 1301-2197; this probe detects all five Mm-*Homer1* transcript variants tv1, tv2, tv3, tv4 and tv5), Mm-*Homer2* (gene ID: 26557; target sequence: 487–1485; this probe detects Mm-*Homer2* tv1, tv2 and tv3) and Mm-*Homer3* (gene ID: 26558; target sequence 105–1237; this probe detects Mm-*Homer3 tv1*). The sections were not counterstained after visualization of hybridization signals. Hybridization signals detected by oligonucleotide probes and the RNAscope method using chromogenic substrates appear as sparse puncta/precipitates when weak to moderate levels of transcripts are expressed in a given tissue/cell [106,107,108] or as aggregated puncta/precipitates when the hybridization signals are robust [61,106,107,108]. For detection of *Homer* mRNAs by in situ hybridization, sections across prenatal and postnatal heads encompassing the brain and other cephalic tissues were processed under the same conditions. This enables comparison of the intensities of hybridization signals between tissues in the same given section.

## 5. Conclusions

In this study, we used immunohistochemistry and in situ hybridization which enabled us to reveal hitherto unknown distribution patterns of Homer1, Homer2 and Homer3 proteins and their encoding genes in developing cephalic tissues and organs. We show that the three Homer family members are expressed in a multitude of developing cephalic tissues and organs, including the forebrain, cochlea, olfactory and respiratory mucosae, taste buds, tooth, salivary glands and bone, and that the Homers are expressed not only in differentiated cells endowed with specialized functions such as secretion, reabsorption of extracellular components or sensory perception, but also in proliferating and differentiating cells during organ growth and morphogenesis. We demonstrate that although, to a large extent, the Homers display overlapping distribution patterns in a given tissue/organ, Homer proteins exhibit similar yet also different subcellular localizations in several cell types within a tissue/organ, suggesting that Homer proteins may have compensatory as well as distinct functions. Homer proteins are known to localize at intracellular puncta in neurons and skeletal muscle [3,5,15,16,17,18]. Our study revealed that Homer proteins are concentrated in intracellular puncta in a wide variety of cephalic non-excitable cells, and that the punctate pattern of Homer distribution is observed in undifferentiated/proliferating cells, differentiating cells as well as in differentiated cells. Finally, our study is the first to show that the vascular endothelium is enriched with Homer3 mRNA and protein and produces Homer1 and Homer2 proteins. Our findings are expected to be of value for future research aiming at unravelling the functions of Homer proteins through cell- and tissue-specific genetic ablation in developing cephalic structures. 

## Supplementary Materials

The following are available online at https://www.mdpi.com/1422-0067/21/4/1264/s1, Figure S1: Assessment of the specificities of Homer probes and Homer antibodies. (A–C) Representative Homer1 (A), Homer2 (B) and Homer3 (C) in situ hybridization data (the signals appear as a brown precipitate) in sections across the hippocampal formation at 12 days postpartum (12 dpp) showing enrichment of Homer1, Homer2 and Homer3 transcripts in the hippocampal CA1, CA1–CA2 and CA3 regions, respectively and expression of the three Homer family members in the dentate gyrus. Note that the transcripts are confined to neuronal somata. Homer3 mRNA is also readily detectable in the endothelium of blood vessels in the hippocampal region and in meninges. (D–F) Representative immunostaining (purple) showing the distribution of Homer1 (D), Homer2 (E) and Homer3 (F) proteins in sections across the hippocampal formation at 12 dpp. The distribution of Homer proteins is consistent with the expression patterns of Homer transcripts, except that the proteins are enriched in neuronal processes. The vascular endothelium exhibits weak Homer1, moderate Homer2 and strong Homer3 immunostaining. D’–F’ are magnified views of the boxed areas in D–F. (G,H). Sections across molar teeth at 1 dpp (G) and 12 dpp (H) processed for immunohistochemistry without the primary antibodies (negative controls) showing absence of immunostaining signals. BV, blood vessels; DG, dentate gyrus; Hl, hilus of the dentate gyrus; MA, maturation-stage ameloblasts; PL, papillary layer; Sb, subiculum; To, tooth. Scale bars: 200 μm (A–F,G) and 50 μm (D’–F’,H). Table S1. Summary of the expression patterns of Homer proteins in murine cephalic tissues and organs.

## Abbreviations

BSA	Bovine serum albumin
GER	Greater epithelial ridge of the developing organ of Corti
IHC	Inner hair cells of the organ of Corti
IP3Rs	Inositol 1,4,5-triphosphate receptors
mGluR1/5	Group I metabotropic glutamate receptors
mTOR	Mammalian/mechanistic target of Rapamycin
NFATc	Nuclear factor of activated T-cells
OHC	Outer hair cells of the organ of Corti
PBS	Phosphate buffered saline
PMCA	Plasma-membrane Ca^2+^-ATPase pump
RANKL	Receptor activator of nuclear factor kappa-B ligand
RT-qPCR	Reverse transcription quantitative polymerase chain reaction
RyRs	Ryanodine receptors
SOCE	Store-operated calcium entry
STIM1/STIM2	Stromal interaction molecules 1 and 2
TRPC	Transient receptor potential canonical
