# Supplementary material for: Distinct and Overlapping Expression Patterns of the Homer Family of Scaffolding Proteins and Their Encoding Genes in Developing Murine Cephalic Tissues
Source: Int J Mol Sci. 2020 Feb 13;21(4):1264. doi: 10.3390/ijms21041264 (PMC7072945; doi:10.3390/ijms21041264)
Supplement: Supplementary file 1 [file ijms-21-01264-s001.pdf]

**Supplementary Figure**

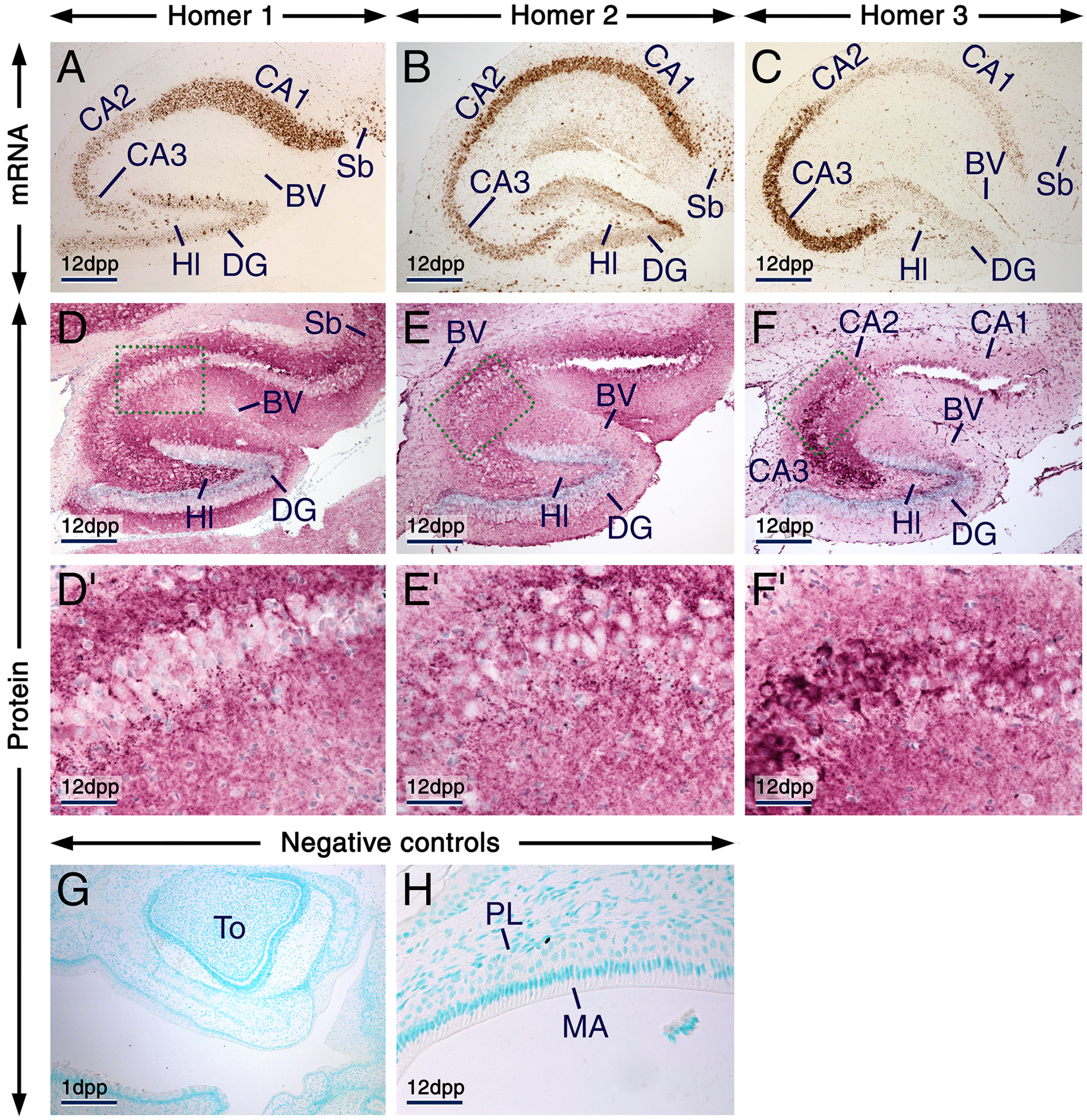


**Figure S1**. **Assessment of the specificities of Homer probes and Homer antibodies.** (**A-C**) Representative *Homer1* (**A**), *Homer2* (**B**) and *Homer3* (**C**) in situ hybridization data (the signals appear as a brown precipitate) in sections across the hippocampal formation at 12 days postpartum (12 dpp) showing enrichment of *Homer1*, *Homer2*, and *Homer3* transcripts in the hippocampal CA1, CA1-CA2, and CA3 regions, respectively, and expression of the three Homer family members in the dentate gyrus. Note that the transcripts are confined to neuronal somata. *Homer3* mRNA is also readily detectable in the endothelium of blood vessels in the hippocampal region and in meninges. (**D-F**) Representative immunostaining (purple) showing the distribution of Homer1 (**D**), Homer2 (**E**) and Homer3 (**F**) proteins in sections across the hippocampal formation at 12 dpp. The distribution of Homer proteins is consistent with the expression patterns of *Homer* transcripts, except that the proteins are enriched in neuronal processes. The vascular endothelium exhibits weak Homer1, moderate Homer2 and strong Homer3 immunostaining. **D′-F′** are magnified views of the boxed areas in **D-F**. (**G,H**). Sections across molar teeth at 1 dpp (**G**) and 12 dpp (**H**) processed for immunohistochemistry without the primary antibodies (negative controls) showing absence of immunostaining signals. BV, blood vessels; DG, dentate gyrus; Hl, hilus of the dentate gyrus; MA, maturation-stage ameloblasts; PL, papillary layer; Sb, subiculum; To, tooth. Scale bars: 200 μm (**A-F**,**G**) and 50 μm (**D′-F′**,**H**).

**Table S1. Summary of the expression patterns of Homer proteins in murine cephalic tissues and organs**

| Tissue/cells | Homer1 | Homer2 | Homer3 |
|---|---|---|---|
| ***Tooth*** | | | |
| Dental placode | ◊ + | *◊ +++ | *◊ +++ |
| Dental mesenchyme | – | * +++ | * +++ |
| *Bud stage* | | | |
| Dental epithelium | *◊ +++ | *◊ +++ | *◊ +++ |
| Dental mesenchyme | *◊ ++ | *◊ + | *◊ + |
| *Cap stage* | | | |
| Dental epithelium | *◊ +++ | *◊ +++ | *◊ ++++ |
| Dental papilla mesenchyme | *◊ ++ | * + | *◊ +++ |
| *Bell stage and advanced stages* | | | |
| Inner dental epithelium | ◊ ++ | ◊ ++ | ◊ +++ |
| Dental papilla mesenchyme | * + | * + | * +++ |
| Differentiating ameloblasts | ◊ ++++ | *◊ ++++ | ◊ ++++ |
| Odontoblasts producing predentin matrix | *◊ ++ | *◊ ++++ | *◊ ++++ |
| Stratum intermedium | ◊ + | ◊ +++ | ◊ ++ |
| Secretory ameloblasts | *◊ ++++ | *◊ ++++ | *◊ +++ |
| Maturation-stage ameloblasts | *◊ ++++ | *◊ ++++ | *◊ +++ |
| Papillary layer | ◊ ++ | *◊ +++ | *◊ +++ |
| Young odontoblasts | ◊ ++ | *◊ ++++ | *◊ +++ |
| Mature odontoblasts | *◊ +++ | *◊ ++++ | *◊ +++ |
| Dental pulp | ◊ + | * ++ | *◊ +++ |
| Hertwig's epithelial root sheath | *◊ ++++ | *◊ ++++ | *◊ ++ |
| ***Forebrain*** | | | |
| Choroid plexus | ‡◊ ++++ | ‡* ++++ | ‡◊ ++++ |
| Hippocampal formation | *◊ ++++ | *◊ ++++ | *◊ ++++ |
| Hypothalamus | *◊ +++ | *◊ ++++ | * ++++ |
| Neocortex | *◊ ++++ | *◊ ++++ | *◊ ++++ |
| Striatum | *◊ ++++ | *◊ ++++ | *◊ ++++ |
| Thalamus | *◊ +++ | *◊ ++++ | *◊ ++++ |
| Hypothalamic neuroepithelium | ‡◊ +++ | ‡*◊ ++++ | ‡*◊ ++++ |
| Ventricular layer | ‡ +++ | ‡ ++++ | ‡ ++++ |
| ***Nasal cavity*** | | | |
| Olfactory epithelium | ‡◊ ++++; †+++ | ‡◊ ++++; †++++ | ‡◊ +++; †+++ |
| Respiratory epithelium | *◊ ++++ | *◊ +++ | *◊ +++ |
| Glands | ‡◊ ++++ | ‡* +++ | ◊ ++ |
| ***Vascular endothelium and cranial nerves*** | | | |
| Vascular endothelium | * ++ | * +++ | * ++++ |
| Cranial nerves | All cranial nerves at E14.5 and postnatally: *+++ | All cranial nerves at E14.5 and postnatally: *++++ | Olfactory nerves at E14.5: *++++. Postnatal olfactory nerves: *+++. Other cranial nerves at E14.5 and postnatally: *+++ |

| | | | |
|---|---|---|---|
| ***Eye*** | | | |
| Retina and lens epithelium | *◊ +++ | *◊ +++ | *◊ ++++ |
| ***Cochlea*** | | | |
| Inner and outer hair cells | ‡*◊ ++++ | ‡*◊ ++++ | *◊ ++ |
| Greater epithelial ridge | ‡*◊ ++++ | ‡* ++ | *◊ +++ |
| Deiter's cells | ◊ ++ | * + | *◊ +++ |
| Claudius's cells | *◊ +++ | *◊ ++ | *◊ +++ |
| Reissner's membrane | *◊ ++ | * + | *◊ +++ |
| Stria vascularis | *◊ +++ | *◊ +++ | *◊ ++++ |
| Spiral ganglion | *◊ ++++ | *◊ +++ | *◊ +++ |
| Basilar membrane | * + | * ++ | * ++++ |
| ***Tongue*** | | | |
| Differentiating lingual epithelium | *◊ ++ | *◊ +++ | *◊ +++ |
| Postnatal lingual epithelium | ◊ ++ | * +++ | * +++ |
| Differentiating taste buds | * ++++ | * +++ | – |
| Postnatal taste buds | * ++++ | *◊ +++ | *◊ ++ |
| Lingual mesenchyme | *◊ +++ | * + | * ++ |
| Developing muscles | ◊ +++ | *◊ ++++ | * ++ |
| Postnatal muscles | § ++++ | ◊ +++ | ◊ ++ |
| ***Submandibular salivary gland*** | | | |
| Embryonic glands | ‡*◊ +++ | *◊ +++ | *◊ +++ |
| Postnatal glands | ‡◊ ++++ | ‡* ++++ | *◊ ++++ |
| ***Secondary palate*** | | | |
| Rugae palatinae | ++++ | *◊ ++ | ◊ +++ |
| Medial epithelial seam | *◊ +++ | *◊ ++ | *◊ +++ |
| ***Alveolar bone*** | | | |
| Osteoblasts | *◊ +++ | *◊ +++ | *◊ ++ |
| Osteocytes | – | * +++ | * +++ |
| Osteoclasts | *◊ +++ | *◊ +++ | *◊ ++++ |

Symbols ‡: enriched in apical membranes and/or in cilia; *: immunostaining of the cytoplasm and plasma membranes; ◊: strong immunostaining in intracellular puncta; †: detectable in subsets of cells, including in olfactory neuron somata and their dendrites and axons; §: banded pattern of immunostaining; very weak (+), weak (++), moderate (+++), and strong (++++) immunolabelling of the cytoplasm and plasma membrane. (–) undetectable immunolabelling. Intracellular Homer-positive puncta may be masked when the overall immunostaining is strong in a given cell.
